# Immune characterization of a Colombian family cluster with SARS-CoV-2 infection

**DOI:** 10.7705/biomedica.5976

**Published:** 2021-10-15

**Authors:** Wbeimar Aguilar-Jiménez, Lizdany Flórez-Álvarez, Daniel S. Rincón, Damariz Marín-Palma, Alexandra Sánchez-Martínez, Jahnnyer Martínez, María Isabel Zapata, John D. Loaiza, Constanza Cárdenas, Fanny Guzmán, Paula A. Velilla, Natalia A. Taborda, Wildeman Zapata, Juan C. Hernández, Francisco J. Díaz, María T. Rugeles

**Affiliations:** 1 Grupo de Inmunovirología, Facultad de Medicina, Universidad de Antioquia, Medellín, Colombia Universidad de Antioquia Grupo de Inmunovirología Facultad de Medicina Universidad de Antioquia Medellín Colombia; 2 Grupo de Salud y Comunidad, Facultad de Medicina, Universidad de Antioquia, Medellín, Colombia Universidad de Antioquia Grupo de Salud y Comunidad Facultad de Medicina Universidad de Antioquia Medellín Colombia; 3 Núcleo de Biotecnología Curauma, Pontificia Universidad Católica de Valparaíso, Valparaíso, Chile Pontificia Universidad Católica de Valparaíso Núcleo de Biotecnología Curauma Pontificia Universidad Católica de Valparaíso Valparaíso Chile; 4 Grupo de Investigaciones Biomédicas Uniremington, Programa de Medicina, Facultad de Ciencias de la Salud, Corporación Universitaria Remington, Medellín, Colombia Corporación Universitaria Remington Grupo de Investigaciones Biomédicas Uniremington, Programa de Medicina Facultad de Ciencias de la Salud Corporación Universitaria Remington Medellín Colombia; 5 Grupo Infettare, Facultad de Medicina, Universidad Cooperativa de Colombia, Medellín, Colombia Universidad Cooperativa de Colombia Grupo Infettare, Facultad de Medicina Universidad Cooperativa de Colombia Medellín Colombia

**Keywords:** Coronavirus infections, inflammation, killer cells, natural, T-lymphocytes, antibodies, neutralizing, infecciones por coronavirus, inflamación, células asesinas naturales, linfocitos T, anticuerpos neutralizantes

## Abstract

**Introduction:**

Immunological markers have been described during COVID-19 and persist after recovery. These immune markers are associated with clinical features among SARS- CoV-2 infected individuals. Nevertheless, studies reporting a comprehensive analysis of the immune changes occurring during SARS-CoV-2 infection are still limited.

**Objective:**

To evaluate the production of proinflammatory cytokines, the antibody response, and the phenotype and function of NK cells and T cells in a Colombian family cluster with SARS-CoV-2 infection.

**Materials and methods:**

Proinflammatory cytokines were evaluated by RT-PCR and ELISA. The frequency, phenotype, and function of NK cells (cocultures with K562 cells) and T-cells (stimulated with spike/RdRp peptides) were assessed by flow cytometry. Anti-SARS-CoV-2 antibodies were determined using indirect immunofluorescence and plaque reduction neutralization assay.

**Results:**

During COVID-19, we observed a high proinflammatory-cytokine production and a reduced CD56^bright^-NK cell and cytotoxic response. Compared with healthy controls, infected individuals had a higher frequency of dysfunctional CD8+ T cells CD38+HLA-DR-. During the acute phase, CD8+ T cells stimulated with viral peptides exhibited a monofunctional response characterized by high IL-10 production. However, during recovery, we observed a bifunctional response characterized by the co-expression of CD107a and granzyme B or perforin.

**Conclusion:**

Although the proinflammatory response is a hallmark of SARS-CoV-2 infection, other phenotypic and functional alterations in NK cells and CD8+ T cells could be associated with the outcome of COVID-19. However, additional studies are required to understand these alterations and to guide future immunotherapy strategies.

## Resumen

**Introducción.:**

Se han descrito diferentes marcadores inmunológicos durante la COVID-19, los cuales persisten incluso después de la convalecencia y se asocian con los estadios clínicos de la infección. Sin embargo, aún son pocos los estudios orientados al análisis exhaustivo de las alteraciones del sistema inmunológico en el curso de la infección.

**Objetivo.:**

Evaluar la producción de citocinas proinflamatorias, la reacción de anticuerpos, y el fenotipo y la función de las células NK y los linfocitos T en una familia colombiana con infección por SARS-CoV-2.

**Materiales y métodos.:**

Se evaluaron las citocinas proinflamatorias mediante RT-PCR y ELISA; la frecuencia, el fenotipo y la función de las células NK (en cocultivos con células K562) y linfocitos T CD8+ (estimulados con péptidos spike/RdRp) mediante citometría de flujo, y los anticuerpos anti-SARS-CoV-2, mediante inmunofluorescencia indirecta y prueba de neutralización por reducción de placa.

**Resultados.:**

Durante la COVID-19 hubo una producción elevada de citocinas proinflamatorias, con disminución de las células NK CD56^*bright*^ y reacción citotóxica. Comparados con los controles sanos, los individuos infectados presentaron con gran frecuencia linfocitos T CD8+ disfuncionales CD38+HLA-DR-. Además, en los linfocitos T CD8+ estimulados con péptidos virales, predominó una reacción monofuncional con gran producción de IL-10 durante la fase aguda y una reacción bifuncional caracterizada por la coexpresión de CD107a y granzima B o perforina durante la convalecencia.

**Conclusión.:**

Aunque la reacción inflamatoria caracteriza la infección por SARS-CoV-2, hay otras alteraciones fenotípicas y funcionales en células NK y linfocitos T CD8+ que podrían asociarse con la progresión de la infección. Se requieren estudios adicionales para entender estas alteraciones y guiar futuras estrategias de inmunoterapia.

COVID-19 is caused by the severe acute respiratory syndrome coronavirus 2 (SARS-CoV-2) ^1^. Currently, more than 212 million people have been infected with the virus worldwide. In Colombia, since the first reported case (March 6, 2020), more than 4.8 million cases have been reported (by August 23, 2021).

SARS-CoV-2 is an enveloped, single-strand, positive-sense RNA virus ^2^. The infection begins with the interaction of the spike protein (S) with the cellular receptor ACE2, highly expressed on lower and upper respiratory tract cells, sites of viral transmission and severe disease development, respectively ^3^. Following infection, a strong inflammatory response is triggered including the high activity of macrophages and neutrophils and their products, reactive oxygen species (ROS), neutrophil extracellular traps (NETs), IL-6, type I IFN, monocyte chemoattractant protein (MCP-1), and human interferon-inducible protein (IP-10), among others ^4^^,^^5^. Systemic inflammation is a key feature, especially in those patients with severe clinical manifestations who exhibit increased levels of IL-6, TNFα, C reactive protein (CRP), and pro-coagulant factors ^6^^,^^7^.

NK cells are pivotal antiviral actors and can be rapidly recruited to different anatomical sites, such as the lungs, to assist the clearance of virus-infected cells. They are subdivided into several subpopulations according to CD56 expression with the differential capacity to produce cytokines or induce apoptosis of target cells ^8^. NK cells are important players in the immune responses in COVID-19 patients by direct elimination of virus-infected cells and the modulation of the systemic inflammatory response ^9^. In addition, some NK cells subpopulations have similar features to adaptive T lymphocytes. In this sense, NKG2C+ NK cells can effectively mediate antibody-dependent effector functions and produce antiviral cytokines after peptide stimulation ^10^^,^^12^. Although studies are still limited in this field, COVID-19 patients exhibit NKG2A overexpression while activation markers are reduced, which suggests NK exhaustion and reduction of their response capacity ^13^.

Subsequent adaptive responses involving B and T cells are observed in infected patients, although humoral response seems to be variable. Detectable IgM and IgG antibodies are produced within days to weeks of the onset of symptoms in most infected individuals without there being a clear association between antibody responses and clinical outcome ^14^. However, some authors have reported stronger antibody response in those patients with severe symptoms ^15^. Both SARS-CoV-2-specific CD8^+^ and CD4^+^ T cells have been reported in the convalescence phase. But lymphopenia is often reported in severe and fatal cases suggesting a key role of adaptive cellular immunity in the control and outcome of SARS-CoV-2 infection ^16^.

SARS-CoV-2-infected patients exhibit variable illness severity including asymptomatic infection, mild or severe disease, and, eventually, death. Although vaccination programs have been progressing adequately in many countries, in others coverage is still very limited. Thus, natural herd immunity should be considered as a potential mechanism against COVID-19, at least in particular community settings (i.e., small or country-side towns). Determining post-infection immunity has a key epidemiological and clinical value and could also have implications in the evaluation of potential immunomodulatory therapies and vaccines. Here we provide a detailed functional and phenotypic characterization of immune parameters in a family cluster with SARS-CoV-2 infection: Two asymptomatic cases and one patient with mild COVID-19. Our findings have important implications for understanding the circumstances under which the immune response could be established during SARS-CoV-2 infection.

## Materials and methods

### Samples

The study on this family cluster (three individuals named JM, A, and JC) was approved by the institutional ethics board of *Universidad de Antioquia* (Colombia) and the informed consent was signed by all the family members in the study.

The clinical data were collected by phone during their follow-up. A nasopharyngeal aspirate from JM was taken on March 25 for SARS-CoV-2 detection by using the Luna® Universal Probe One-Step RT-qPCR Kit (New England Biolabs) and the CDC RT-PCR oligos and probes protocol ^17^. Furthermore, viral isolation was performed from the nasopharyngeal sample and the virus was amplified for the experiments ^18^.

Blood samples were obtained on March 25 (acute phase), April 14 (convalescent phase), and May 28 (recovery phase) from JM. Additionally, samples from A and JC were collected on May 28. Serum was obtained by centrifugation from anticoagulant-free and plasma from EDTA-blood and then stored at -80°C until their use for antibody testing by immunofluorescence and cytokine quantification by ELISA. Peripheral blood mononuclear cells (PBMCs) were isolated by EDTA blood centrifugation on a density gradient with Ficoll-Histopaque® (Sigma-Aldrich, St. Louis, MO, USA) and frozen in liquid nitrogen until immune assays. Also, fresh PBMC from three healthy donors age- and sex-matched with the family members were collected at the time of donors and included in some of the assays.

### Quantification of inflammatory molecules by RT-PCR

Total RNA was isolated from PBMCs using the Direct-zol™ RNA Miniprep (Zymo Research., CA, USA), then quantified using a Multiskan SkyHigh Microplate Spectrophotometer (Thermo Fisher Scientific, Hanover, MD, USA), and cDNA synthesis was done with 150 ng of total RNA using the High-Capacity cDNA Reverse Transcription Kit (Applied Biosystems^™^, CA, USA) following the manufacturers’ recommendations. The expression of IL-1β, IL-6, IL-8, and TNFα, as well as of the phosphoglycerate kinase (PGK) used as reference gene, was quantified by real-time RT-PCR using the Maxima SYBR^®^ Green qPCR master mix (Thermo Fisher Scientific, Hanover, MD, USA), and a CFX-96 Real-Time thermal cycler (Bio-Rad, Hercules, CA, USA) as previously described ^19^. Oligos and thermal conditions are shown in supplementary material 1. Real-time RT-PCR analysis was conducted using the CFX Maestro 1.1 Software (Bio-Rad).

### ELISA

Plasma levels of IL-1β, IL-6, and IL-8 were quantified in duplicate by ELISA using commercial kits from eBioscience (Vienna, Austria), BD Biosciences (Franklin Lakes, NJ), and Biolegend (San Diego, CA, USA), respectively, following the manufacturers’ instructions.

### NK cells phenotype by flow cytometry

Briefly, 1 x10^6^ PBMCs were stained using monoclonal antibodies against CD45, CD56, CD3, CD57, and NKG2C for 25 minutes in the dark (antibody details are shown in supplementary material 2). Then, cells were washed twice with phosphate-buffered saline (PBS) (Lonza, Rockland, ME, USA), suspended in paraformaldehyde 2%, and acquired using LSR Fortessa^™^ (BD Biosciences, San Jose, CA, USA). The data were analyzed using FlowJo software, version 10.5.3 (FlowJo, LLC, Oregon, USA).

### NK cytotoxicity assay

PBMCs were thawed and left in culture with RPMI (Sigma-Aldrich) supplemented with 10% fetal bovine serum (FBS) (Sigma-Aldrich) for 24 h before the experiments. K562 cells were used as targets ^20^. Prior to culturing, 1×10^6^ K562 cells were stained with 0.1 mM eFluor™ 670 (Thermo Fisher Scientific) in PBS for 10 min at 37°C. Then, PBMCs were co-cultured with K562 cells in round-bottomed tubes at a 10:1 ratio in 300 µl of RPMI with 10% FBS for 4 h at 37°C and 5% CO_2_.

After incubation, cells were stained with propidium iodide (PI) and DIOC- 6 (both from Thermo Fisher Scientific) for 15 min in the dark. PI and DIOC-6 were used to evaluate the integrity of the cell and mitochondrial membranes, respectively. K562 cells cultured in the absence of PBMCs were used as spontaneous death control, which was conducted for every assay. Spontaneous death control had to be lower than 15% for the experiment to be valid. The cytotoxicity percentage was adjusted based on spontaneous death control.

### Anti-SARS-CoV-2 antibodies

*Indirect Immunofluorescence assay.* Sera samples of all family members were subjected to indirect immunofluorescence assay (IFA) to determine the titer of specific antibodies against SARS-CoV-2.

Vero E6 monolayers grown in 75 cm^2^ flasks were infected or not with a Colombian SARS-CoV-2 isolate (SARS-CoV-2/human/Medellin/UdeA1/2020) ^20^ at a multiplicity of infection (MOI) of 0.001 in 2 ml of DMEM supplemented with 2% FBS for 1 h; after virus adsorption, the inoculum was removed and replaced with fresh medium. Cells were detached with scrapper 48 h.p.i and resuspended in 3 ml PBS. The cell suspension was spotted in 10 well-immunofluorescence slides (20 ul/well) and dried overnight. Afterward, the slides were fixed by immersion in acetone for 30 min and stored at 4°C until used.

Two-fold dilutions of sera in PBS (1:5 to 1:80) were added to infected and uninfected cells (20 ul each) and incubated in a moist chamber at 37°C for 30 minutes, then washed twice by immersion in PBS for 5 min with slow shaking and then allowed to dry. After, 20 ul of anti-human IgG-FITC (Sigma- Aldrich) diluted 1:16 in PBS were added to each well and incubated in a moist chamber at 37°C for 30 min followed by two washes. The slides were mounted with anti-fade reagent Fluosaver (Calbiochem) and coverslips and visualized in an Axio Vert.A1 (ZEISS, Oberkochen, Germany) fluorescent microscope at 400x magnification by three different researchers. Antibody titer was reported as the last dilution where fluorescence was clearly observed in infected cells but no so at the same dilution in non-infected cells. Experiments requiring virus handling were done in a biosafety level 3 laboratory.

*Plaque reduction neutralization test.* Neutralizing antibodies in the sera of infected individuals were tested using a 50% plaque reduction neutralization test (PRNT50) with Vero E6 cells. Briefly, Vero E6 cells (1.1 × 10^5^ cells per well) were seeded into the 24-well tissue culture plates the day before infection. On the next day, 200 plaque-forming units of SARS-CoV-2 were incubated with or without serially diluted heat-inactivated sera (56°C, 30 min) in a total volume of 200 μl in microcentrifuge tubes for 60 min at 37°C and 5% CO_2_. Next, the mixtures were added to Vero E6 monolayers and incubated at 37°C for 60 min. Then, the inoculum was removed and 1ml of the semisolid medium (containing 1.5% of carboxymethyl cellulose 2% fetal bovine serum, 1% streptomycin, and DMEM) was added and cultured at 37°C for 72 h. Afterwards, the semisolid media was removed and monolayers were washed twice with PBS. Finally, monolayers were fixed and stained with 1% crystal violet/4% formaldehyde for 30 min and washed twice with PBS. A 50% reduction in plaque count (PRNT50) in two independent assays conducted by two different researchers was used as the neutralizing endpoint. The inhibition percentage was calculated based on the number of plaques on infection control wells. To discard toxicity, a serum control in uninfected cells per individual was also included in the assays. Experiments requiring virus handling were done in a biosafety level 3 laboratory.

*T cell bBasal activation phenotype.* Phenotypic analysis of PBMCs was done from frozen samples. T cells percentages and basal CD4^+^ and CD8^+^ T cell activation levels were assessed by flow cytometry. Cells were incubated with monoclonal antibodies against surface molecules CD4, CD8, HLA- DR, CD69, and CD38 for 25 min. Then, cells were permeabilized and fixed using the Foxp3/Transcription Factor Staining kit (Thermo Fisher Scientific), and intracellular staining for 25 min with antibodies against Ki-67 and CD3 was performed (see antibody details in supplementary material 2). At least 100,000 events were acquired on an LSR Fortessa^™^ (BD) and analyzed with the FlowJo (BD) software.

### *Peptide synthesis, purification, and characterization for functional evaluation of CD8* ^*+*^ *T cells*

Spike and RdRp peptide pools are listed in supplementary material 3A and their location at the protein is visualized in a 3D model in supplementary material 3B-C. Spike and RdRp peptide pools are based on the PDB structures 6VSB and 6M71, respectively. The peptides in the spike peptide pool are located on the surface area (the exposed region) of the glycoprotein ^21^. The peptides in the RdRp are sites involved in the protein folding which is part of the interaction of the complex formed by nsp12-nsp7-nsp8 ^22^. During this analysis, we did not have information on the patient’s HLA-I, therefore, PBMCs were stimulated with peptides located in different sites of the proteins.

Peptides were synthesized using a Liberty Blue™ automated microwave peptide synthesizer (CEM Corp., Matthews, NC, USA) following a standard 9-fluorenylmethoxycarbonyl (Fmoc)/tert-butyl (tBu) protocol as previously described ^23^. Peptides were hydrolyzed with TFA/TIS/Water/DOT (proportion 92.5:2.5:2.5:2.5) and purified by RP-HPLC; their purity and molecular weights were confirmed by electrospray mass spectrometry (ESI-MS), they were then lyophilized, reconstituted in sterile water (1 mg/ml), and stored at −20ºC until used.

*Peptide stimuli and flow cytometry analysis of CD8* ^*+*^ *T cells.* Thawed PBMCs were cultured at a density of 4 ×10^6^ cells/ml in RPMI-1640 medium supplemented with 10% FBS, 100 U/ml penicillin, 100 µg/ml of streptomycin, and 2 mM L-glutamine (complete medium; all from Sigma-Aldrich). The cells were stimulated with 1 ug/ml of both anti-CD28 (clone: CD28.2, eBioscience^™^), and anti-CD49d (clone: 9F10, eBioscience^™^) functional grade antibodies plus 10 μg/ml peptide pools (spike and RdRp). Cells stimulated only with anti-CD28 and anti-CD49d antibodies were used as negative controls. Stimulation with 1 ug/ml of peptide pools of Staphylococcal enterotoxin B (SEB) (Sigma-Aldrich) was used as a positive control. All groups of cells were incubated for 12 h at 37˚C in 5% CO_2_ in the presence of 10 μg/ml Brefeldin A and 7μM Monensin (both from Thermo Fisher) ^24^.

After incubation, PBMCs were harvested, washed with PBS (Sigma- Aldrich), and stained with conjugated antibodies against surface molecules CD3 and CD8, and with Fixable Viability Dye eFluor^™^ 506 (Thermo Fisher Scientific) at 4°C for 30 min. Afterwards, the cells were fixed and permeabilized with Foxp3 Fixation/Permeabilization Buffer (Thermo Fisher Scientific). Then, the following antibodies were added: anti-IL-2, anti-granzyme B, anti-perforin, anti-IFN- γ, anti-TNF-α, and anti-IL-10, and incubated at 4°C for 30 minutes (antibody details are shown in supplementary material 2). Cells were acquired in an LSR Fortessa™ flow cytometer using the BD FACSDiva^™^ software v 8.0.1 (BD). At least 100,000 events of the CD3^+^ T cells were recorded. To evaluate the polyfunctional response, a Boolean gating strategy was performed to create a full array of possible expression combinations of effector molecules up to 64 response patterns from the CD8^+^ T-cell gate. The data was reported after background subtraction (subtraction of the negative control) and correction. Background correction was defined according to the number of functions. For subsets with one and two functions, a level of 0.05% was considered as the threshold for a positive response. For the subsets with three and four function responses, the threshold was defined as 0.005%. In the case of five and six function responses, the threshold was defined as 0.0005%. Subsequently, polyfunctionality was visually represented using the SPICE software v5.35 ^25^.

### Ethics approval and consent to publication

All the necessary ethical considerations were observed. The study was approved by the ethics committee at *Universidad de Antioquia*. All research protocols adjusted to the principles of the Declaration of Helsinki. The donors gave their written informed consent for the obtention of the clinical information to be published.

## Results

### Cluster description

The mild COVID19 case corresponded to a 59-year-old man residing in Medellín (JM). He had comorbidities such as hypertension, diabetes mellitus, and hypercholesterolemia, all of which were treated and controlled at the time of infection. He had traveled to southwest Europe (Portugal and Spain) with his family (including his two sons, A and JC, 24 and 35 years old, respectively) and returned to Colombia on March 12, 2020. Four days later, JM showed the first symptoms including headache, high dorsal pain, sored pharynx, mild coughing, and fever (38.2^º^C). In the following days, JM developed drowsiness, anorexia, and anosmia. The dorsal pain was moving downward in his back and he was treated with paracetamol.

On March 19, nasopharyngeal aspirates were taken from all family members. The RT-PCR tests were positive for JM, A, and JC. On March 25, a blood sample was drawn from JM (JM-1) and an additional nasopharyngeal aspirate was taken for viral isolation (SARS-CoV-2/human/Medellin/ UdeA1/2020). On this date, the SARS-CoV-2 RNA was detected at the cycle threshold (Ct) 19 in JM’s nasopharyngeal aspirate indicating a high viral load even after nine days of the onset of the symptoms.

**Figure 1:**
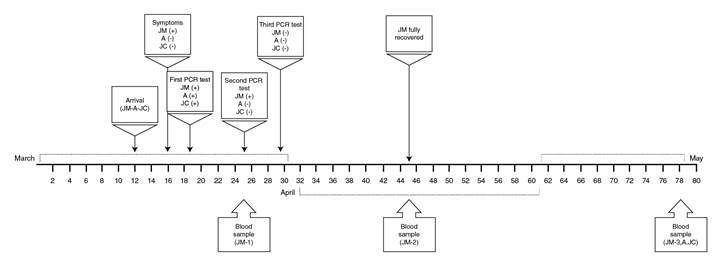
Timeline of clinical evolution and sampling of a family cluster with SARS-CoV-2 infection. The family, composed by JM, his wife (not infected), and his sons A and JC likely acquired the SARS-CoV-2 infection during a trip to southwest Europe, but only JM was symptomatic. The time points at which samples were taken from the family members are indicated in the figure.

In the following days, JM had prolonged asthenia and poorly defined discomfort in his back, but no clinical complications were observed. On March 30, the RT-PCR for SARS-CoV-2 was negative for JM, A, and JC, and on April 14 a convalescent blood sample was taken for antibody and immune testing (JM-2). By April 19, JM was fully recovered (phone follow-up) and on May 28, blood samples from all family members (JM-3, A, and JC) were taken for immune characterization. The timeline is described in figure 1.

### Increased inflammatory cytokines during the acute symptomatic phase of infection

**Figure 2:**
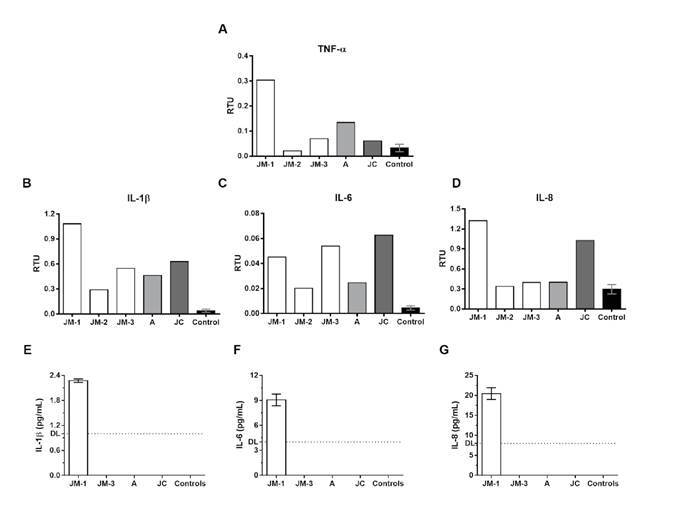
Proinflammatory cytokine expression. The mRNA expression of TNF-α (A), IL-1β (B), IL-6 (C), and IL-8 (D) was evaluated by real time RT-PCR in PBMCs from the family members and healthy controls (n=3). The results are presented as relative transcripts units (RTU) normalized over the expression of the PGK reference gene. Protein levels of IL-1β (E), IL-6 (F), and IL-8 (G) were evaluated by ELISA in plasma from the family members and healthy controls. DL: detection limit

Higher mRNA levels of inflammatory cytokines IL-1β, IL-6, IL-8, and TNF-α were observed during early symptomatic infection (JM-1) compared to healthy controls. These cytokine levels decreased in the convalescent phase (second and third sampling time) except for IL-6, whose levels increased again in the third sampling time (figure 2A-D).

Regarding the asymptomatic but infected donors (A and JC), JC had a different profile and exhibited higher mRNA expression of IL-6 and IL-8 compared to A and JM-3 at the same point of infection. Finally, the expression of IL-1β and IL-6 in the three family members remained higher than in the healthy controls even after two months of negative SARS-CoV-2 by RT-PCR.

ELISA quantification of IL-1β, IL-6, and IL-8 in plasma was undetectable in JM-3, A, and JC, as well as in the healthy controls, but they were detected during the acute symptomatic infection in JM (JM-1) (figure 2E-G).

### NK cells phenotype

**Figure 3:**
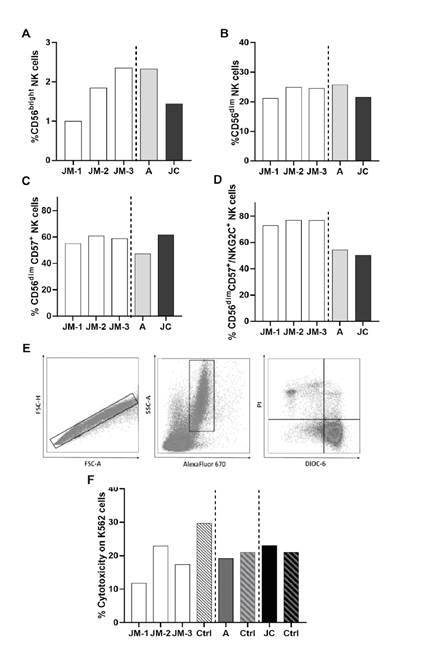
NK cell phenotype and cytotoxicity. Percentage of cytokine-producer CD56bright (A), cytotoxic CD56^dim^ (B), mature CD56^dim^ CD57+(C), and adaptive CD56^dim^ CD57+ NKG2C+ (D) NK cells subsets in JM, A, and JC. JM-1, JM-2, JM-3 correspond to three different JM sampling times (see figure 1 for details on sampling times). The cytotoxicity was expressed as the percentage of K562 DIOC-6- PI+ cells: (E) Representative gate of target cells (K562) selected by eFluor670 expression. (F) Percentage of cytotoxicity for each subject compared with the respective age- and sex-paired control

We evaluated the main two NK cell subpopulations, CD56^bright^ (representing the cytokine-producer subset, mainly IFN- γ), and CD56^dim^ (cytotoxic cells), as well as terminally differentiated NK cells identified by the expression of the maturation marker CD57. Adaptive NK cells, which are terminally differentiated memory-like NK cells with high expression of activating receptor NKG2C, were also included in the analysis. The percentage of CD56^bright^ NK cells increased over time in JM (figure 3A). The frequency of the other NK cell subsets in JM’s samples did not differ considerably in any of the points in time (figure 3B-D). When the other family members were analyzed, a lower frequency of CD56^bright^ NK cells was observed in JC compared to JM-3 and A (figure 3A). Interestingly, the percentage of CD56^dim^, CD57^+^, and NKG2C^+^ adaptive NK cells was lower in A and JC compared to JM at all evaluated times (figure 3D).

### Decreased NK activity during the symptomatic SARS-CoV-2 infection

The cytotoxic capacity was evaluated by co-cultures of the K562 cell line with PBMCs from each donor. A representative scheme of the cytotoxicity assessment is shown in figure 3E. Compared with the controls, decreased cytotoxic NK cells activity was observed in JM, with the lowest activity occurring during the symptomatic acute infection (JM-1) (figure 3F). Although NK cell activity seemed to increase in the convalescent phase (JM-2), it remained lower than the age- and sex-paired control (figure 3F). No differences were observed in asymptomatic subjects (A and JC) compared to their age- and sex-paired healthy controls.

### Anti-SARS-CoV-2 antibodies

*Indirect immunofluorescence.* Specific antibodies against SARS-CoV-2 were detected in family members’ sera by IFA with serum dilutions ranging from 1:5 to 1:80. JM showed titers of specific anti-SARS-CoV-2 antibodies of 1:5 at the first sample time and increased to 1:20 at the third sample time. A and JC’s titers were 1:5 and 1:10, respectively.

*Neutralizing antibodies.* Once the specific anti-SARS-CoV-2 antibodies were detected in the family cluster sera, the titers of the neutralizing antibodies were established by PRNT50 at 1:20, 1:200, and 1:2000 serum dilutions on VeroE6 cells.

The second sample from JM (JM-2) showed a plaque reduction of 51.3% at 1:200 dilution while his third sample (JM-3) showed a slight decrease in the titer (40.4% at 1:200 dilution). A’s titer was close to 1:200 (46.6% plaque reduction) while JC’s was <1:20 (48.3% plaque reduction) (supplementary material 4).

### T cells activation phenotype

The percentage of CD4^+^ and CD8^+^ T cells expressing HLA-DR, CD38, and CD69 activation markers, as well as the Ki-67 proliferation marker, was measured on PBMCs obtained from all family members.

**Figure 4:**
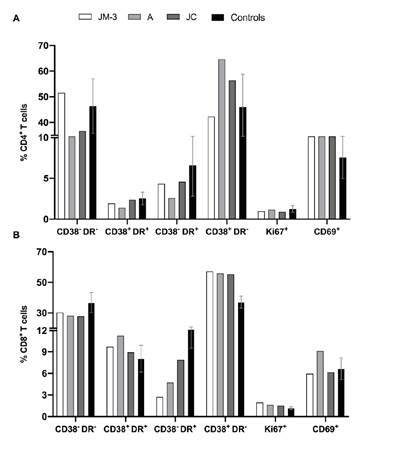
Activation phenotype of T cells. We explored the co- expression of CD38 and HLA-DR, as well as the percentages of the proliferation marker Ki-67 and the activation marker CD69 in CD4+ (A) and CD8+ (B) cells from the family members in the convalescent phase and healthy controls (n=3).

We found a slight increase in the percentages of CD38^+^ HLA-DR- CD4^+^ T cells in A (64.6%) and JC (56.4%) compared to JM-3 (42.3%) (figure 4A). In contrast, CD69+ CD4+ T cell frequency was similar for JM-3 (14.5%), A (15%), and JC (16%), but such percentages were higher compared to those observed in healthy controls (media ± SD; 7.6 ± 2.6%) (figure 4A).

On the other hand, similar percentages of CD38^+^ HLA-DR^-^ CD8^+^ T cells were found for JM-3 (57.2%), A (55.9%), and JC (55.3%), which were higher compared to those observed in healthy controls (37.1 ± 4.0%) (figure 4B). On the contrary, the percentage of CD38^-^ HLA-DR^+^ CD8^+^ T cells was lower in family members during the recovery phase compared to healthy controls (15.2 ± 5.7%), and much lower in JM-3 (2.8%) compared to A (4.7%) and JC (7.9%) (figure 4B).

Regarding the Ki-67 proliferation marker, no differences were observed in CD4^+^ T cells nor CD8^+^ T cells between family members and healthy controls.

### Frequency and functional profile of antigen-specific SARS-CoV-2 CD8+ T cells

**Figure 5:**
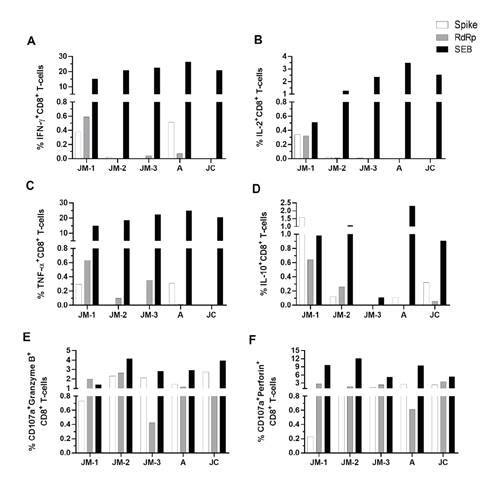
CD8^+^ T cell response to SARS- CoV-2 peptide pools. Frequency of CD8^+^ T cells positive for cytokines IFN-γ **(A)**, IL-2 **(B)**, TNF-α **(C)**, and IL-10 **(D)**, as well as degranulation marker CD107a along with granzyme B **(E)** and perforin **(F)**. Each bar represents the CD8^+^ T cell response in an individual in response to spike (white bars), RdRp (gray bars) peptide pools or SEB (black bars) after subtraction of the negative control.

The functional profile of antigen-specific SARS-CoV-2 CD8^+^ T cells was established through the evaluation of cytokine and cytotoxic molecule expression in response to spike and RdRp peptide pools (supplementary material 3). SARS-CoV-2 specific cytokine-producing CD8+ T cells in response to both peptide pools were observed mainly at the first follow-up point in time for JM when he was still symptomatic; lower or undetectable frequencies were observed at days 30 (JM-2) and 73 (JM-3) after the onset of the symptoms, as well as in asymptomatic individuals (A and JC) 73 days after diagnosis (figure 5A-D). While SARS-CoV-2 specific IL-10-producing CD8^+^ T cells were more frequent in JC than A and JM-3, A had a higher percentage of SARS-CoV-2 specific IFN-γ- and TNF-α-producing CD8+ T cells compared to A and JM-3, mainly in response to spike peptides (figure 5A-D).

Regarding the capacity of CD8^+^T cells to produce *de novo* granzyme B and perforin (evaluated by the coexpression of CD107a with each cytotoxic molecule) in response to both peptide pools, CD107a^+ g^ranzymeB^+^ and perforin^+^ CD8^+^ T cells were detectable up to 73 days after the onset of symptoms with their highest at day 30 (figure 5E, F). Both asymptomatic individuals (A and JC) developed a cytotoxic response which remained for up to 73 days after diagnosis (figure 5E-F), but no differences were observed among the family members.

**Figure 6:**
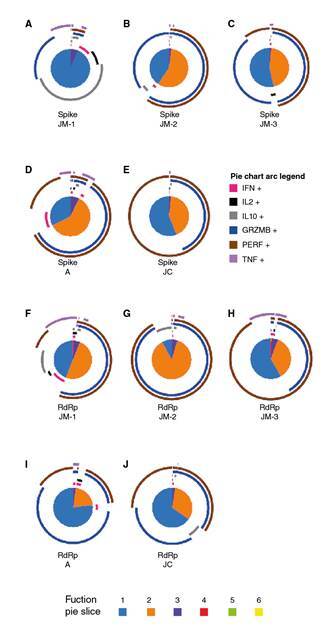
Polyfunctional CD8+ T cell response to SARS-CoV-2 spike and RdRp peptides. Pie charts represent the polyfunctional profile of CD8+ T cells in response to the spike in JM’s follow-up (A-C), and asymptomatic A (D), and JC (E), as well as the polyfunctional profile of CD8+ T cells in response to RdRp in JM’s follow-up (F-H) and asymptomatic A (I) and JC (J). Arcs depict molecule make-up within the pie slice. The combination of functions evaluated is indicated by pie chart colors.

Additionally, we evaluated the quality of SARS-CoV-2-CD8^+^ T cell response considering that the polyfunctionality of CD8^+^ T cells has been associated with better control of viral infection. During JM’s follow up, the response against spike peptides changed from a predominant monofunctional response, at the expense mainly of IL-10 (JM-1), to a higher proportion of bifunctional cells where CD107a^+^granzymeB^+^ and CD107a^+^perforin^+^ profile was dominant (JM-2 and JM-3) (figure 6A, B and C).

With regard to the response to spike peptides in asymptomatic individuals about 73 days post-diagnosis, A exhibited a major proportion of the predominant pattern of bifunctional responses dominated by CD107a^+^granzymeB^+^ and CD107a^+^perforin^+^ CD8^+^ T cells compared to JM-3 and JC. However, the monofunctional response in asymptomatic individuals consisted of perforin whereas in JM-3 it was mainly granzyme B (figure 6C-E).

Concerning RdRp peptides, we observed a high proportion of cells exhibiting two functions with cytotoxic profile followed by the monofunctional responses at the expense of all the cytokines evaluated when the individual was symptomatic (JM-1) (figure 6F). However, after 30 days of follow up, the highest proportion of two functions had a cytotoxic profile (figure 6G) whereas at day 73, monofunctional responses predominated but still at the expense of perforin (figure 6H). Besides, donor A exhibited a major proportion of CD8^+^ T cells with monofunctional responses compared to JM-3 and JC (figures 6H-J). Again, the bifunctional profile was dominated by a cytotoxic profile in both asymptomatic individuals (figures 6I-J).

Altogether, these results suggest an association between the development of the cytotoxic capacity of antigen-specific SARS-CoV-2-CD8^+^ T cells and mild disease or even asymptomatic infection.

## Discussion

COVID-19 infection has been characterized for highly variable disease manifestations ^26^^,^^27^ and immune response elicited ^28^^,^^29^. Here, we evaluated the immunological profile of a family cluster including a mild COVID-19 case (JM) and two asymptomatic infections (A and JC). Interestingly, a more robust immune response characterized by higher IFN-γ-producer CD56^bright^ NK cells, higher neutralizing antibodies titer, and higher percentage of SARS-CoV-2 specific IFN-γ- and TNF-α-producing CD8^+^ T cells were observed in donor A compared to JC, both with common genetic and demographic characteristics, infected around the same time, and most likely with the same viral strain. It is important to point out that A was 11 years younger than JC, although both are considered young adults, and this difference should not account for the observed results.

Despite neutralizing antibodies have been correlated with protection against SARS-CoV-2 infection ^30^^,^^31^, these are not strictly required to clear the SARS-CoV-2 infection as observed in the asymptomatic neutralizing- negative subject JC, and as previously suggested for young people (aged 16-30) ^32^. Similarly, donor A showed low neutralizing antibody levels, which raises the following question: What is the protection factor in individuals without significant humoral responses? The symptomatic donor JM had a reduction in neutralizing antibody titers in the convalescent period, as previously reported ^33^.

Inflammation is a hallmark of COVID19 severe cases in contrast with lower immune activation that seemingly characterizes asymptomatic cases ^33^. Proinflammatory cytokines measured by PCR and ELISA in JM peaked during the acute infection and decreased in the convalescent phase, at least in plasma, to levels near those found in healthy controls. These results are in line with previous reports, especially those for IL-6 and IL-8 ^34^^,^^35^. In contrast, a previous report did not find differences in IL-1b or TNF-a levels when comparing infected versus uninfected donors ^34^. Small differences were observed in the convalescent phase among JM (recovered from symptomatic infection), A, and JC (recovered from asymptomatic infection) suggesting that proinflammatory cytokines are restored to baseline levels after the virus is cleared.

Antiviral NK cells also seem to be altered during acute SARS-CoV-2 infection since the frequency of IFN-γ-producer CD56^bright^ NK cells and cytotoxic activity against K562 cells was lower during this phase in JM-1 compared to JM-2, and JM-3 (30 and 73 days after symptoms onset, respectively), and to A and JC in the convalescent phase.

No changes in the adaptive NKG2C^+^ NK cells frequency as shown by effectively mediated antibody-dependent effector functions and cytokine production ^10^^,^^11^ in the hematopoietic stem cell transplantation setting, the presence of donor activating KIRs (aKIRs) were observed during JM’s follow- up. We want to highlight that the frequency of this cell subpopulation was higher in JM, an older subject, compared to donors A and JC. Indeed, the frequency of this NK cells subset has been reported to increase with age ^36^.

In contrast to cytokines and NK cells, the frequency of activated CD69^+^ CD4^+^ and CD38^+^ CD8^+^ T cells remained higher in the convalescent phase, regardless of infection symptomatic or asymptomatic nature, compared to healthy controls. This aspect would relate to persistent hyperactivation of the T cell profile despite viral clearance or due to a SARS-CoV-2 specific response ^37^. Indeed, whereas cytokine-producing CD8^+^ T cells were mainly observed during the symptomatic acute infection in JM, and lower or absent during the convalescent phase in JM, A, and JC, CD107a^+^ granzymeB^+^ and perforin^+^ CD8^+^ T cells remained detectable up to 73 days after diagnosis in all family members. These results are similar to a previous report showing lower cytokine expression on CD8^+^ T cells in the mild disease compared with severe cases, although the activation markers remained highly expressed suggesting a lack of T cell response contraction ^37^.

During JM’s follow-up, we observed a higher frequency of SARS-CoV-2- specific-CD8^+^T cells evaluated through the production of cytokines, which declined after the resolution of the infection as previously described in a preprint ^38^. Importantly, we observed that spike-CD8^+^T cells in JM produced lower amounts of granzyme and perforin during the acute phase of the infection in comparison to the following time points, which suggests a reduced cytotoxic capacity of specific-CD8^+^ T cells in COVID-19 patients consistent with previous reports ^38^^,^^39^.

We also evaluated the polyfunctionality of CD8^+^ T cells, a correlate of immune protection, including not only the production of cytokines, as in other reports, but also the production of cytotoxic molecules. The polyfunctionality of SARS- CoV-2-specific-CD8^+^ T cells in JM during the acute phase of the infection was mainly monofunctional as has been suggested for symptomatic patients ^38^. However, after 30 days of the onset of the symptoms, we observed an increase in the frequency of bifunctional cells (secreting granzyme B and perforin), which declined after 73 days of symptoms onset. In this sense, one study showed a decrease in the functional diversity of T cells in patients compared with healthy controls ^40^. However, other authors have reported polyfunctionality, particularly by CD4^+^ T cells expressing TNF-a, IL-2, and IL-17 ^41^.

The frequency of specific-CD8^+^ T cells capable of producing cytotoxic molecules in asymptomatic individuals was similar to that observed in JM during the convalescent phase. Indeed, the polyfunctional response of asymptomatic individuals was mainly mono- and bifunctional with prevalent production of cytotoxic molecules. This suggests that the cytotoxic capacity of spike-specific- CD8^+^ T cells may be correlated to protection in SARS-CoV-2 infection.

Our results suggest a strong inflammatory response during mild COVID-19 disease with a reduction of CD56^bright^ NK cells subset during the symptomatic phase and lower cytotoxic response of these cells. Compared with the asymptomatic infection and uninfected individuals, we observed a higher frequency of dysfunctional CD38^+^ HLA-DR^-^ CD8^+^ T cells subpopulation during the symptomatic phase. Additionally, CD8^+^ T cells had a predominant monofunctional response with high IL-10 production in mild COVID-19 but a bifunctional response dominated by CD107a^+^granzymeB^+^ and CD107a+perforin+ CD8^+^ T cells in asymptomatic infection suggesting an important role of spike-specific-CD8^+^ T cells. However, our study had a limited sample size and few SARS-CoV-2 peptides were analyzed, so additional studies comparing immune profiles in individuals with different progression patterns could provide information to develop immunotherapy strategies.

## References

[1] Li Q, Guan X, Wu P, Wang X, Zhou L, Tong Y (2020). Early transmission dynamics in Wuhan, China, of novel coronavirus-infected pneumonia. N Engl J Med.

[2] Chen Y, Liu Q, Guo D (2020). Emerging coronaviruses: Genome structure, replication, and pathogenesis. J Med Virol.

[3] Ortiz ME, Thurman A, Pezzulo AA, Leidinger MR, Klesney-Tait JA, Karp PH (2020). Heterogeneous expression of the SARS-Coronavirus-2 receptor ACE2 in the human respiratory tract. EBioMedicine.

[4] Chu H, Chan JFW, Wang Y, Yuen TT, Chai Y, Hou Y (2020). Comparative replication and immune activation profiles of SARS-CoV-2 and SARS-CoV in human lungs: An ex vivo study with implications for the pathogenesis of COVID-19. Clin Infect Dis.

[5] Huang C, Wang Y, Li X, Ren L, Zhao J, Hu Y (2020). Clinical features of patients infected with 2019 novel coronavirus in Wuhan, China. Lancet.

[6] Yuan X, Tong X, Wang Y, Wang H, Wang L, Xu X (2020). Coagulopathy in elderly patients with coronavirus disease 2019. AGING Med.

[7] Ponti G, Maccaferri M, Ruini C, Tomasi A, Ozben T (2020). Biomarkers associated with COVID-19 disease progression. Crit Rev Clin Lab Sci.

[8] Taborda NA, Hernández JC, Montoya CJ, Rugeles MT (2014). Natural killer cells and their role in the immune response during human immunodeficiency virus type-1 infection. Inmunologia.

[9] Alrubayyi A (2020). NK cells in COVID-19: Protectors or opponents?. Nat Rev Immunol.

[10] Della Chiesa M, Sivori S, Carlomagno S, Moretta L, Moretta A (2015). Activating KIRs and NKG2C in viral infections: Toward NK cell memory?. Front Immunol.

[11] Schlums H, Cichocki F, Tesi B, Theorell J, Beziat V, Holmes TD (2015). Cytomegalovirus infection drives adaptive epigenetic diversification of NK cells with altered signaling and effector function. Immunity.

[12] Florez-Alvarez L, Blanquiceth Y, Contreras K, Ossa-Giraldo AC, Velilla PA, Hernandez JC (2020). NK cell activity and CD57+/NKG2Chigh phenotype are increased in men who have sex with men at high risk for HIV. Front Immunol.

[13] Zheng M, Gao Y, Wang G, Song G, Liu S, Sun D (2020). Functional exhaustion of antiviral lymphocytes in COVID-19 patients. Cell Mol Immunol.

[14] Sun B, Feng Y, Mo X, Zheng P, Wang Q, Li P (2020). Kinetics of SARS-CoV-2 specific IgM and IgG responses in COVID-19 patients. Emerg Microbes Infect.

[15] Wang Y, Zhang L, Sang L, Ye F, Ruan S, Zhong B (2020). Kinetics of viral load and antibody response in relation to COVID-19 severity. J Clin Invest.

[16] Huang I, Pranata R (2020). Lymphopenia in severe coronavirus disease-2019 (COVID-19): Systematic review and meta-analysis. J Intensive Care.

[17] Centers for Disease Control and Prevention-CDC Real-time RT-PCR Primers and Probes for COVID-19.

[18] Feria MG, Taborda NA, Hernandez JC, Rugeles MT (2018). HIV replication is associated to inflammasomes activation, IL-1β, IL-18 and caspase-1 expression in GALT and peripheral blood. PLoS ONE.

[19] Florez-Alvarez L, Blanquiceth Y, Ramirez K, Ossa-Giraldo AC, Velilla PA, Hernández JC (2020). NK cell activity and CD57+/NKG2Chigh phenotype are increased in men who have sex with men at high risk for HIV. Front Immunol.

[20] Diaz FJ, Aguilar-Jimenez W, Florez-Alvarez L, Valencia G, Laiton-Donato K, Franco-Munoz C (2020). Aislamiento y caracterizacion de una cepa temprana de SARS-CoV-2 durante la epidemia de 2020 en Medellin, Colombia. Biomedica.

[21] Cai Y, Zhang J, Xiao T, Peng H, Sterling SM, Walsh RM (2020). Distinct conformational states of SARS-CoV-2 spike protein. Science.

[22] Gao Y, Yan L, Huang Y, Liu F, Zhao Y, Cao L (2020). Structure of the RNA-dependent RNA polymerase from COVID-19 virus. Science.

[23] Luna OF, Gómez J, Cardenas C, Albericio F, Marshall SH, Guzman F (2016). Deprotection reagents in Fmoc solid phase peptide synthesis: Moving away from piperidine?. Molecules.

[24] Perdomo-Celis F, Velilla PA, Taborda NA, Rugeles MT (2019). An altered cytotoxic program of CD8 + T-cells in HIV-infected patients despite HAART-induced viral suppression. PLoS ONE.

[25] Roederer M, Nozzi JL, Nason MC (2011). SPICE: Exploration and analysis of post-cytometric complex multivariate datasets. Cytom Part A.

[26] Shi Y, Wang G, Cai X, Deng J, Zheng L, Zhu H (2020). An overview of COVID-19. J Zhejiang Univ Sci B.

[27] Ge H, Wang X, Yuan X, Xiao G, Wang C, Deng T (2020). The epidemiology and clinical information about COVID-19. Eur J Clin Microbiol Infect Dis.

[28] Garcia LF (2020). Immune response, inflammation, and the clinical spectrum of COVID-19. Front Immunol.

[29] Azkur AK, Akdis M, Azkur D, Sokolowska M, van de Veen W, Bruggen MC (2020). Immune response to SARS-CoV-2 and mechanisms of immunopathological changes in COVID-19. Allergy Eur J Allergy Clin Immunol.

[30] Ni L, Ye F, Cheng ML, Feng Y, Deng YQ, Zhao H (2020). Detection of SARS-CoV-2-specific humoral and cellular immunity in COVID-19 convalescent individuals. Immunity.

[31] Duan K, Liu B, Li C, Zhang H, Yu T, Qu J (2020). Effectiveness of convalescent plasma therapy in severe COVID-19 patients. Proc Natl Acad Sci USA.

[32] Wang X, Guo X, Xin Q, Pan Y, Hu Y, Li J (2020). Neutralizing antibody responses to severe acute respiratory syndrome coronavirus 2 in coronavirus disease 2019 in patients and convalescent patients. Clin Infect Dis.

[33] Long QX, Tang XJ, Shi QL, Li Q, Deng HJ, Yuan J (2020). Clinical and immunological assessment of asymptomatic SARS-CoV-2 infections. Nat Med.

[34] Qin C, Zhou L, Hu Z, Zhang S, Yang S, Tao Y (2020). Dysregulation of immune response in patients with Coronavirus 2019 (COVID-19) in Wuhan, China. Clin Infect Dis.

[35] Han H, Ma Q, Li C, Liu R, Zhao L, Wang W (2020). Profiling serum cytokines in COVID-19 patients reveals IL-6 and IL-10 are disease severity predictors. Emerg Microbes Infect.

[36] Le Garff-Tavernier M, Beziat V, Decocq J, Siguret V, Gandjbakhch F, Pautas E (2010). Human NK cells display major phenotypic and functional changes over the life span. Aging Cell.

[37] Kang CK, Han GC, Kim M, Kim G, Shin HM, Song KH (2020). Aberrant hyperactivation of cytotoxic T-cell as a potential determinant of COVID-19 severity. Int J Infect Dis.

[38] Payen D, Cravat M, Maadadi H, Didelot C, Prosic L, Dupuis C (2020). A longitudinal study of immune cells in severe COVID-19 patients. Front Immunol.

[39] Mazzoni A, Salvati L, Maggi L, Capone M, Vanni A, Spinicci M (2020). Impaired immune cell cytotoxicity in severe COVID-19 is IL-6 dependent. J Clin Invest.

[40] Zheng HY, Zhang M, Yang CX, Zhang N, Wang XC, Yang XP (2020). Elevated exhaustion levels and reduced functional diversity of T cells in peripheral blood may predict severe progression in COVID-19 patients. Cell Mol Immunol.

[41] De Biasi S, Meschiari M, Gibellini L, Bellinazzi C, Borella R, Fidanza L (2020). Marked T cell activation, senescence, exhaustion and skewing towards TH17 in patients with COVID-19 pneumonia. Nat Commun.

